# Genome sequence of *Bacillus* sp. strain BAU-SS-2023, isolated from nasal swab of cattle in Bangladesh

**DOI:** 10.1128/mra.01249-24

**Published:** 2025-02-27

**Authors:** Md. Abdur Rahman, Jahangir Alam, Farah Zereen, Md. Golzar Hossain, Sukumar Saha

**Affiliations:** 1 Department of Microbiology and Hygiene, Bangladesh Agricultural University 54492 , Mymensingh, Bangladesh; 2 Department of Animal Production, Gono Bishwabidyalay 421872 , Dhaka, Bangladesh; 3 Animal Biotechnology Division, National Institute of Biotechnology 563134 , Dhaka, Bangladesh; 4 Department of Microbiology, Gono Bishwabidyalay 421872 , Dhaka, Bangladesh; Portland State University, Portland, Oregon, USA

**Keywords:** *Bacillus *sp., nasal swab, cattle, Bangladesh

## Abstract

We report the genome sequence of the *Bacillus* sp. strain BAU-SS-2023, isolated from nasal swabs of cattle in Bangladesh. The strain was isolated using brain heart infusion (BHI) broth and blood agar media. The genome was 9,162,285 bp, 32.4% G+C content, 9,145 coding sequences, 6 rRNAs, 73 tRNAs, and 9 noncoding RNAs.

## ANNOUNCEMENT

Bacillus species are Gram-positive, spore-forming, rod-shaped, motile bacteria that can survive in various environmental settings (1). *Bacillus* sp. BAU-SS-2023 strain was identified through colony characteristics and draft genome sequence itself along with *Pasteurella multocida* serotype B2 from nasal swab samples of clinically suspected hemorrhagic septicemia showing signs of fever, restlessness, nasal discharge, and progressive respiratory distress of nine crossbred dairy cattle at Trishal (90.0242560°E, 24.2235986°N), Mymensingh, Bangladesh. The nasal swab samples were separately inoculated into brain heart infusion (BHI) broth (2) and kept under constant shaking at 180 rpm for 48 hours at 37°C in a shaking incubator. Then, the enriched samples were inoculated separately into bovine blood agar media and incubated overnight at 37°C (3). Small, round, white, opaque, glistening, and non-hemolytic colony characteristics resembling *Bacillus* spp. were observed on blood agar from three different samples. Among them, a single colony of BAU-SS-2023 strain was selected, picked, and purified three times by repeated streaking onto bovine blood agar. Then, a single colony from that blood agar was inoculated into BHI broth. Genomic DNA was extracted from the same culture broth at sterile conditions using the Monarch Genomic DNA Purification Kit (New England Biolabs Inc.) and was sequenced on the Illumina MiSeq platform, with a maximum read length of 2 × 300 bp. First, genomic DNA was fragmented into 400–550 bp pieces using the M220 Focused-ultrasonicator (Covaris Ltd., Brighton, UK), and the Nextera XT DNA Library LT kit (Illumina, San Diego, CA, USA) was used to construct the DNA sequencing library. With a sequence depth of 50× coverage, the Illumina sequencing yielded 626,056 paired-end reads in total.

The raw sequence data underwent quality control using the FastQC (version 0.11.9) tool. Following this, adapter sequence trimming was performed with the fastp (version 0.23.2) (4). With the paired-end reads in hand, we employed Unicycler (version 0.4.8) tools which use SPADES for genome assembly (5). This resulted in a genome 9,162,285 bp long consisting of 85 contigs with N50 length 210,837 and GC content 32.4%. Post-assembly, genome annotation was carried out using the NCBI prokaryotic annotation pipeline PGAP (6), which predicted 9,233 genes, including 9,145 CDSs (coding sequences), 88 RNAs, 6 rRNAs, 73 tRNAs, and 9 noncoding RNAs (ncRNAs).

**Fig 1:**
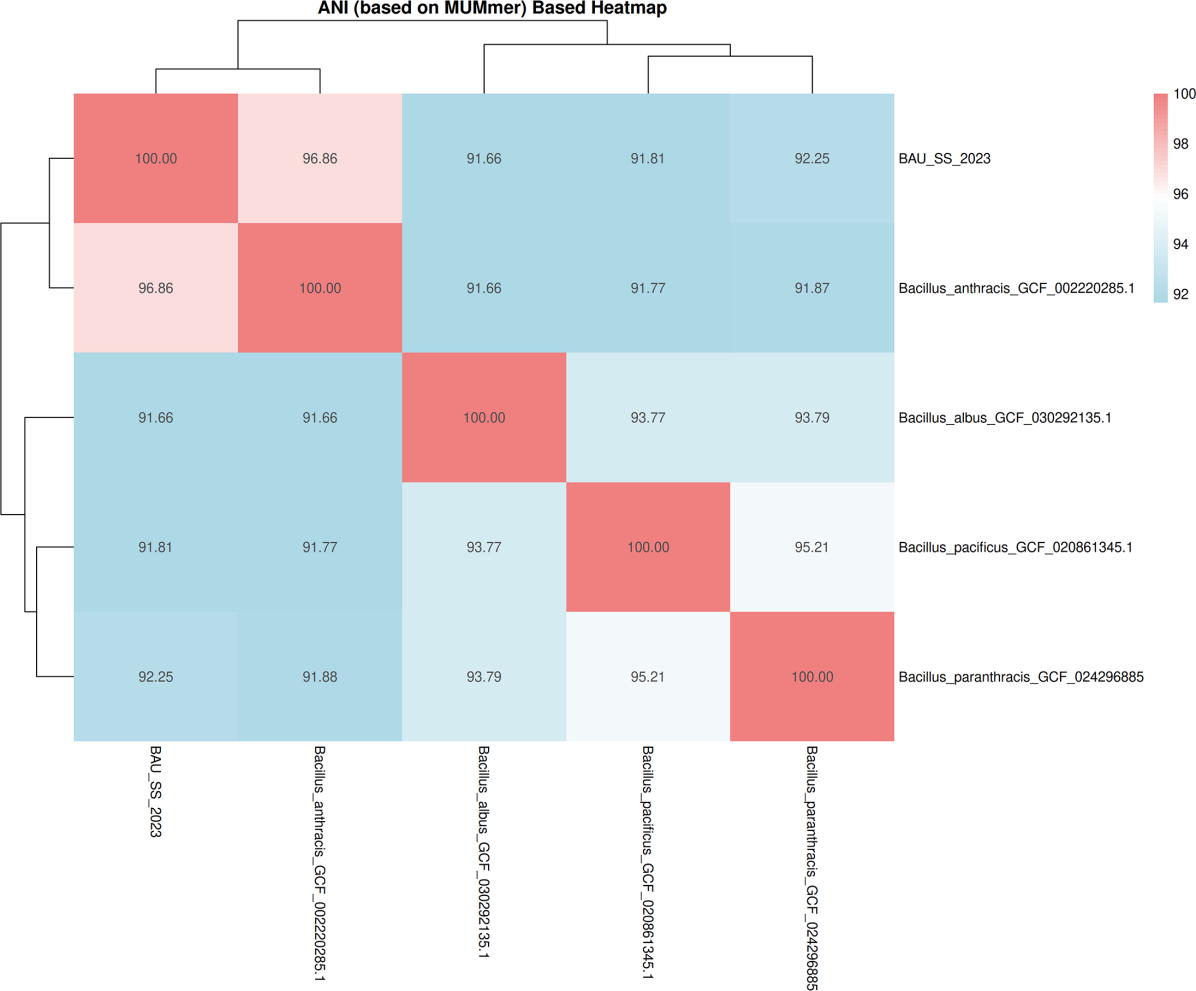
Heatmap of *Bacillus* spp. Average nucleotide identity (ANI) was calculated using JSpeciesWS Online Service (https://jspecies.ribohost.com/) using ANIm (MUMmer-based method). Here, above cutoff (>95%) and below cutoff (<95%) nucleotide similarity is the indication of similar species.

The contigs of the assembled genome were subjected to screening for virulence properties in the Virulence Factor Database-VFDB (7). The antibiotic resistance database was used to find plausible gene candidates linked to antimicrobial resistance (8, 9) and found three antibiotic resistance genes such as VanR-M_1, VanZ-F_1, and vat (E)_10 (10). This screening was performed using Abricate (version 0.5), with parameter cutoffs of 90% coverage and 95% nucleotide identity (11). Average nucleotide identity was calculated with JSpeciesWS and visualized on a clustered heatmap (12), in which about 91.66%–96.86% nucleotide identity was found with different *Bacillus* spp. (Fig. 1). Default parameters were used for all software.

## Data Availability

The genome sequence for *Bacillus* sp. strain BAU-SS-2023 has been deposited in GenBank under the accession number JAWMSB000000000, BioProject number PRJNA1030950, BioSample number SAMN37920875 and SRA number SRX24403959.

## References

[1] Nicholson WL, Munakata N, Horneck G, Melosh HJ, Setlow P. 2000. Resistance of Bacillus endospores to extreme terrestrial and extraterrestrial environments. Microbiol Mol Biol Rev 64:548–572. doi:10.1128/MMBR.64.3.548-572.2000 10974126 PMC99004

[2] Brain Heart Infusion Broth. 2017. Brain heart infusion broth liquid medium for the cultivation of various fastidious organisms and detection of staphylococci.

[3] Lu Z, Guo W, Liu C. 2018. Isolation, identification and characterization of novel Bacillus subtilis. J Vet Med Sci 80:427–433. doi:10.1292/jvms.16-0572 29367516 PMC5880821

[4] Chen S, Zhou Y, Chen Y, Gu J. 2018. Fastp: an ultra-fast all-in-one FASTQ preprocessor. Bioinformatics 34:i884–i890. doi:10.1093/bioinformatics/bty560 30423086 PMC6129281

[5] Wick RR, Judd LM, Gorrie CL, Holt KE. 2017. Unicycler: resolving bacterial genome assemblies from short and long sequencing reads. PLOS Comput Biol 13:e1005595. doi:10.1371/journal.pcbi.1005595 28594827 PMC5481147

[6] Tatusova T, DiCuccio M, Badretdin A, Chetvernin V, Nawrocki EP, Zaslavsky L, Lomsadze A, Pruitt KD, Borodovsky M, Ostell J. 2016. NCBI prokaryotic genome annotation pipeline. Nucleic Acids Res 44:6614–6624. doi:10.1093/nar/gkw569 27342282 PMC5001611

[7] Chen L, Zheng D, Liu B, Yang J, Jin Q. 2016. VFDB 2016: hierarchical and refined dataset for big data analysis--10 years on. Nucleic Acids Res 44:D694–D697. doi:10.1093/nar/gkv1239 26578559 PMC4702877

[8] Jia B, Raphenya AR, Alcock B, Waglechner N, Guo P, Tsang KK, Lago BA, Dave BM, Pereira S, Sharma AN, Doshi S, Courtot M, Lo R, Williams LE, Frye JG, Elsayegh T, Sardar D, Westman EL, Pawlowski AC, Johnson TA, Brinkman FSL, Wright GD, McArthur AG. 2017. CARD 2017: expansion and model-centric curation of the comprehensive antibiotic resistance database. Nucleic Acids Res 45:D566–D573. doi:10.1093/nar/gkw1004 27789705 PMC5210516

[9] Liu B, Zheng D, Jin Q, Chen L, Yang J. 2019. VFDB 2019: a comparative pathogenomic platform with an interactive web interface. Nucleic Acids Res 47:D687–D692. doi:10.1093/nar/gky1080 30395255 PMC6324032

[10] Letunic I, Bork P. 2019. Interactive Tree Of Life (iTOL) v4: recent updates and new developments. Nucleic Acids Res 47:W256–W259. doi:10.1093/nar/gkz239 30931475 PMC6602468

[11] Seemann T. 2019. ABRicate: mass screening of contigs for antimicrobial resistance or virulence genes. Available from: https://github.com/tseemann/abricate

[12] Richter M, Rosselló-Móra R, Oliver Glöckner F, Peplies J. 2016. JSpeciesWS: a web server for prokaryotic species circumscription based on pairwise genome comparison. Bioinformatics 32:929–931. doi:10.1093/bioinformatics/btv681 26576653 PMC5939971

